# Photocatalytic upcycling of plastic waste coupled with CO_2_ reduction to high-value chemicals

**DOI:** 10.1016/j.isci.2026.117589

**Published:** 2026-09-24

**Authors:** Yueyang Zhang, Yufei Gui, Guanqun Han

**Affiliations:** 1 School of Advanced Energy, Sun Yat-sen University, Shenzhen, Guangdong 518107, P.R. China

**Keywords:** plastic waste, photocatalysis, photoreforming, CO_2_ reduction, high-value chemicals

## Abstract

Plastic waste imposes a severe burden on the ecological environment, and CO_2_ emissions have led to frequent extreme weather events. Photoreforming of plastics coupled with CO_2_ reduction has attracted widespread attention by utilizing waste plastics both as electron donors and, via intermediate CO_2_ release in some cascaded pathways, as secondary carbon sources. Photogenerated holes directly oxidize and degrade plastics into high-value chemicals, while the retained electrons drive CO_2_ reduction to gaseous fuels such as CO and CH_4_, thereby enabling concurrent plastic upcycling and CO_2_ utilization. This review synthesizes the core differences between conventional CO_2_ reduction and plastic photoreforming CO_2_ reduction, clarifies the dual role of plastics, and summarizes relevant performance studies across three system configurations, including conventional photocatalysis, plastic-coupled photoreforming, and photoelectrocatalytic integration. Finally, it discusses key challenges and outlines future research directions for advancing next-generation plastic photoreforming CO_2_ reduction technology toward practical implementation.

## Graphical abstract

**Figure:**
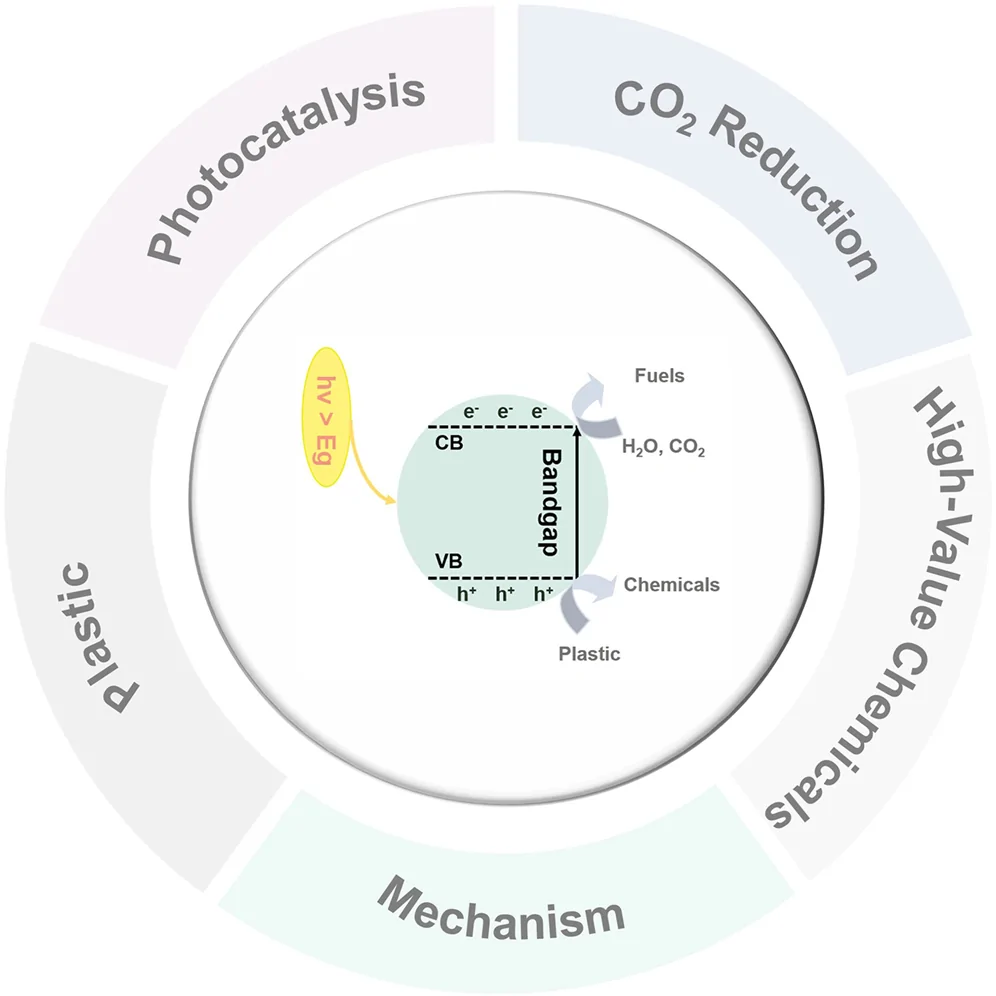

## Teaser

Catalysis; Electrochemistry; Energy storage; Energy materials

## Introduction

Plastics, including polyethylene (PE), polypropylene (PP), polystyrene (PS), polylactic acid (PLA), and PE terephthalate (PET), have become indispensable in modern industry and daily life due to their low cost, low density, lightweight nature, and stable chemical resistance.^1^^,^^2^^,^^3^^,^^4^^,^^5^ However, their widespread use has also created a severe environmental burden.^6^^,^^7^^,^^8^^,^^9^ As shown in Figure 1A, global plastic production has increased continuously from 370.6 million metric tons in 2018 to 430.9 million metric tons in 2024, according to the Plastic Europe Industry report (Plastics—The Facts 2025). Among these, fossil–fuel–derived polymers, such as PE, PP, and PET, account for the largest share (Figure 1B). These synthetic macromolecules exhibit extremely slow natural degradation, typically requiring 2–5 centuries to fully mineralize in the environment, thereby imposing a persistent ecological threat that is difficult to mitigate over the long term.^10^^,^^11^^,^^12^^,^^13^^,^^14^

**Figure 1:**
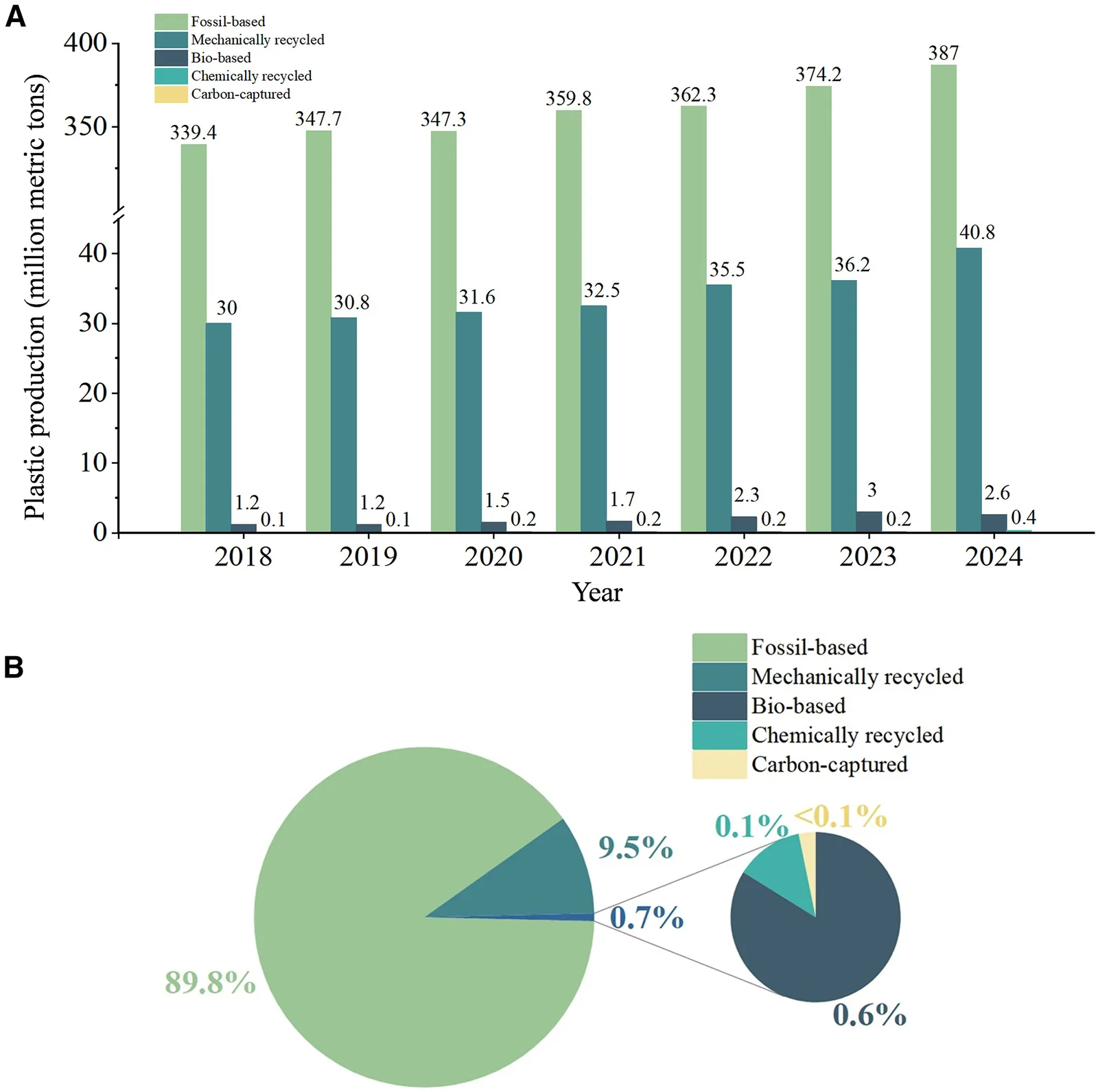
Overview of the global plastics market (A) 2018–2025 plastics growth rate. (B) The composition of plastics.

In parallel with plastic accumulation, anthropogenic CO_2_ emissions since the Industrial Revolution have driven atmospheric CO_2_ volume fractions to historically unprecedented levels, contributing to global climate change and extreme weather events.^15^^,^^16^^,^^17^^,^^18^ Photocatalytic CO_2_ reduction has emerged as a promising strategy to address these challenges, as it utilizes solar energy to convert CO_2_ into carbon-based fuels (e.g., CH_4_, CO, and CH_3_OH) and value–added chemicals, effectively storing intermittent solar energy in chemical bonds while mitigating greenhouse gas emissions.

Traditional CO_2_ reduction technologies generally fall into three categories: thermocatalytic hydrogenation, electrochemical reduction, and bioconversion. Thermocatalytic hydrogenation uses H_2_ as a reducing agent to convert CO_2_ over metal-based catalysts, but typically requires high temperatures and pressures, leading to substantial energy consumption, high maintenance costs, and considerable carbon emissions throughout the process.^19^^,^^20^^,^^21^ Electrocatalytic CO_2_ reduction relies on external electricity to drive cathodic reduction coupled with water oxidation at the anode. However, the competing hydrogen evolution reaction at the cathode severely lowers the Faradaic efficiency (FE) for CO_2_ reduction.^22^^,^^23^ Bioconversion employs algal or microbial enzymatic systems to convert CO_2_ into organic compounds, yet the long fermentation cycles limit its scalability for industrial flue-gas treatment.^24^^,^^25^^,^^26^ In contrast, photocatalytic technology is driven by solar energy, an abundant and renewable resource, making it a particularly attractive alternative. Upon light absorption, a semiconductor photocatalyst generates electron-hole pairs, from which the electrons reduce CO_2_, while the holes oxidize water to O_2_. Nevertheless, rapid charge recombination necessitates the addition of large amounts of organic sacrificial agents, which not only raises operational costs but also introduces secondary pollution. More importantly, none of the above approaches can simultaneously address the two intertwined global challenges, plastic waste accumulation and CO_2_ emissions, as they focus exclusively on CO_2_ conversion without utilizing or degrading plastic waste.^27^

Scientists have demonstrated that the low-molecular-weight organic compounds produced by plastic decomposition can serve as electron donors in photocatalytic systems, eliminating the need for external sacrificial agents. Photogenerated holes directly oxidize and degrade plastics such as PE, PET, and PP (Figure 2A). Depending on the oxidation depth, two distinct pathways are involved: (1) partial oxidation of plastics into high-value liquid chemicals (e.g., alcohols, aldehydes, and carboxylic acids), coupled with reduction of externally supplied CO_2_, and (2) deep mineralization of plastics to release CO_2_ as an internal carbon source, which is then photocatalytically reduced to gaseous fuels. In both pathways, the retained photogenerated electrons drive the CO_2_ reduction, while the rapid consumption of holes by plastic oxidation suppresses carrier recombination. Figure 2B illustrates the conversion paths of different plastics.

**Figure 2:**
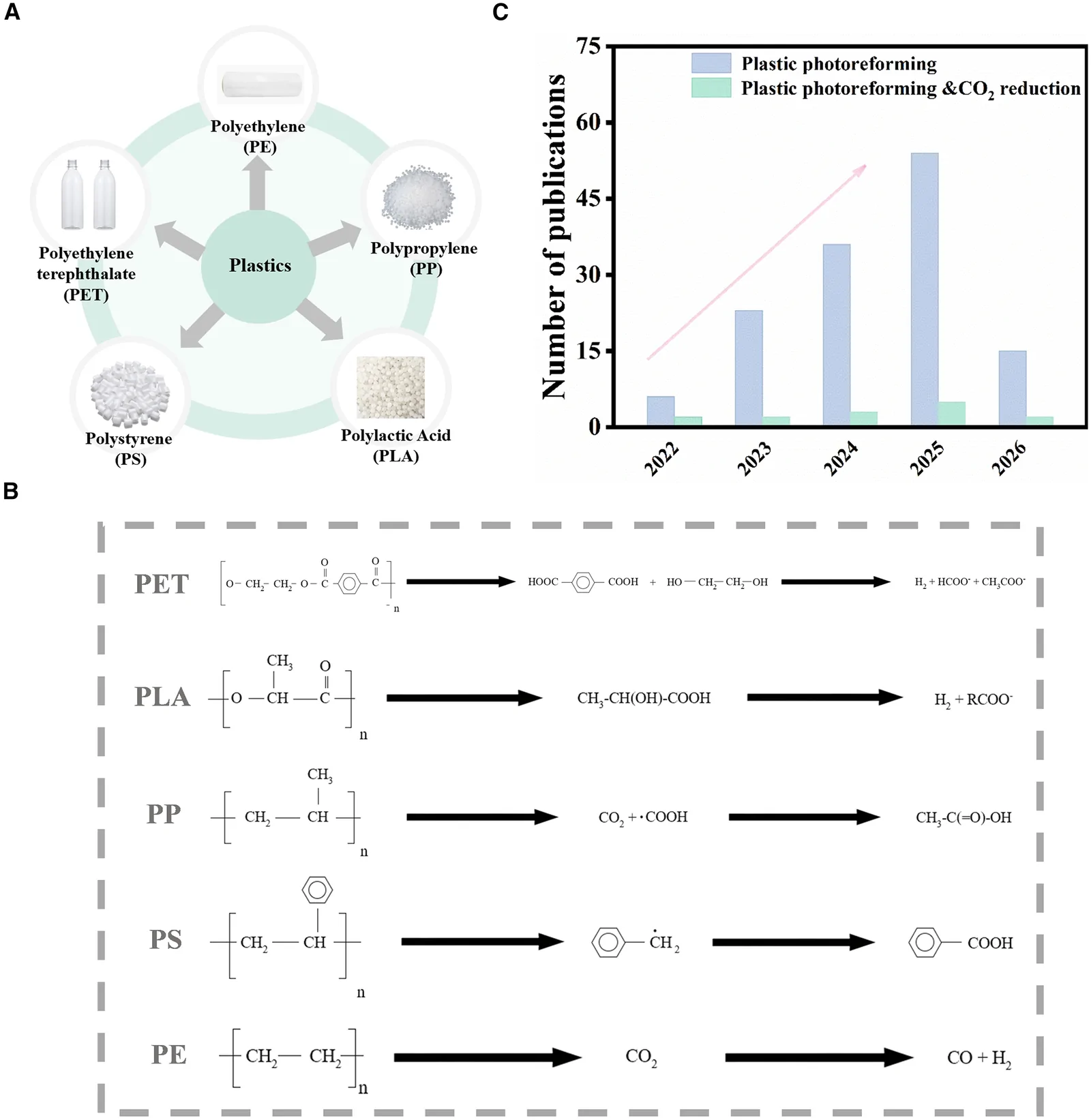
Plastic photoreforming: feedstocks, reaction pathways, and research trends (A) Types of plastics. (B) Conversion pathways of typical plastics with key intermediates and main oxidation/reduction products. (C) Number of publications on plastic photoreforming and plastic photoreforming coupled CO_2_ reduction in recent years.

Furthermore, although photocatalytic plastic degradation technology can convert solid plastics under low-temperature, low-pressure conditions, thereby eliminating solid waste pollution, CO_2_ is produced as the final product, which remains a greenhouse gas, and carbon resources cannot be fully recycled. In contrast, photoreforming can simultaneously convert CO_2_ into fuels or chemicals by taking CO_2_ as a carbon source. Furthermore, photocatalytic degradation systems rely solely on photogenerated holes to drive oxidative chain breaking; photogenerated electrons are not utilized, so a synergistic redox dual-function pathway cannot be established. In contrast, photoreforming provides a stable source of protons and electrons through plastic oxidation, which can significantly reduce the overpotential for CO_2_ reduction while simultaneously improving carrier separation efficiency. Finally, degradation products are often complex mixtures of oxygen-containing small molecules that are difficult to selectively separate and reuse, whereas photoreforming enables selective control over C_1_/C_2_ products, offering greater process controllability.^28^^,^^29^^,^^30^^,^^31^

Owing to these advantages, the number of related research publications has shown a steady annual increase (Figure 2C). For example, Feng et al. comprehensively analyzed the latest advances and innovations in combining photocatalysis with waste plastic recycling, highlighting new pathways for producing renewable hydrogen and resource recycling.^32^ Zhang et al. summarized the latest advances in photocatalytic waste plastic recycling, focusing on the development of highly efficient photocatalysts, an in-depth understanding of reaction mechanisms, and the design or optimization of reaction systems.^33^ However, these existing reviews mainly emphasize H_2_ production or general degradation mechanisms, and limited reviews have systematically addressed the specific integration of CO_2_ reduction with plastic photoreforming. To fill this gap, this review provides a focused overview of photocatalytic CO_2_ reduction coupled with plastic waste valorization. We systematically compare the fundamental differences between conventional CO_2_ reduction and plastic-coupled photoreforming, particularly clarifying the dual role of plastics as both electron donors and secondary carbon sources. Key advances in three system configurations, namely conventional photocatalysis, plastic-coupled photoreforming, and photoelectrocatalytic integration, are critically assessed, with special attention to the production of high-value liquid chemicals from plastic oxidation and gaseous fuels from CO_2_ reduction. Finally, we identify major bottlenecks in catalyst design, feedstock pretreatment, and process scale-up, and outline prospective research directions toward practical carbon-neutral applications.

## The evolution of plastic photocatalytic upgrading: From CO_2_ reduction to waste recycling

### Timeline of CO_2_ reduction from plastic photoreforming

A chronological overview of key milestones in the development of plastic photoreforming coupled with CO_2_ reduction is presented in Figure 3. As early as 1983, the first demonstration that photocatalysis could enable resource recovery from plastic waste was reported. UV-light-driven TiO_2_ was shown to catalyze C-C bond cleavage in PE and PS polymers, yielding carboxylic acids and low-molecular-weight organic compounds.^34^ In the following decades, efforts primarily focused on photodegradation of various polymers under UV or visible light irradiation,^35^^,^^36^^,^^37^ but these studies mainly targeted complete mineralization to CO_2_ and H_2_O, rather than product valorization. A conceptual breakthrough occurred in 2019, when Reisner et al. demonstrated that sunlight could drive a CdS/CdO_x_ photocatalyst to simultaneously produce H_2_ fuel from water and oxidize plastic waste into valuable organic chemicals (e.g., acetates and formates) under ambient conditions.^38^ This work coined the term “photoreforming” for this dual-functional process and shifted the research paradigm from degradation toward upcycling. Subsequent studies extended this approach to real-world plastic feedstocks.^39^^,^^40^^,^^41^^,^^42^^,^^43^^,^^44^ For instance, Kang et al. established a quantitative concentration-product relationship for PET photoreforming, demonstrating that product selectivity could be precisely tuned by adjusting substrate concentration, which provided foundational data for closed-loop PET recycling.^40^ More recently, Zhang et al. reported a single-atom Pd/TiO_2_ catalyst that selectively converts PE plastic bags into ethylene and propionic acid at room temperature and atmospheric pressure, achieving exceptionally high selectivity (98.8%) toward propionic acid.^41^ These advances collectively demonstrate the growing potential of plastic photoreforming for simultaneous waste valorization and CO_2_ utilization.

**Figure 3:**
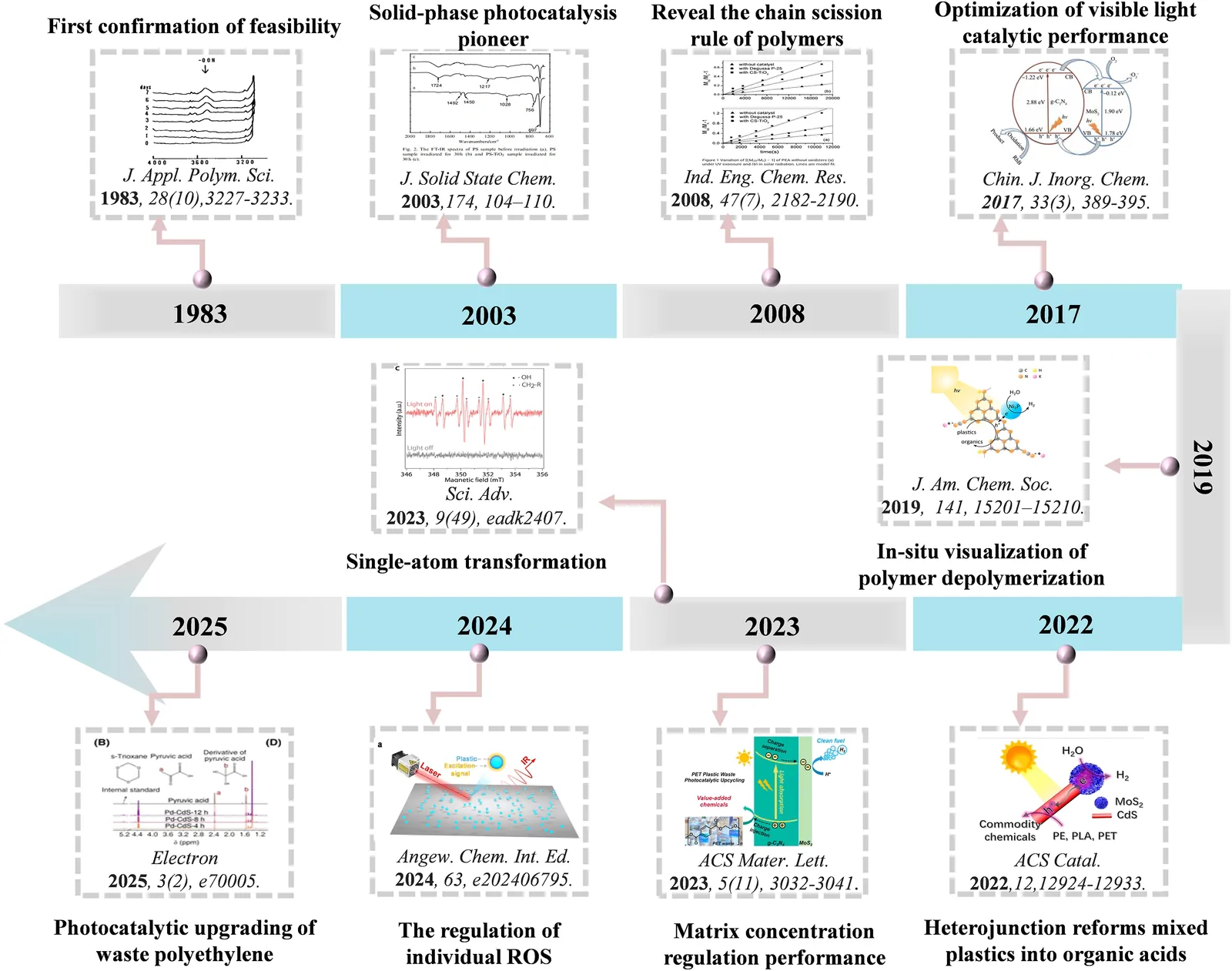
Timeline of CO_2_ reduction from plastic photoreforming Reproduced from Otocka et al.,^34^ copyright@1983, Elsevier. Reproduced or adapted with permission from Shang et al.,^35^ copyright@2003, Elsevier. Reproduced or adapted with permission from Marimuthu and Madras,^36^ copyright@2008, American Chemical Society. Reproduced or adapted with permission from Meng-Qiu et al.,^37^ reproduced under the terms of the CC-BY license. Reproduced or adapted with permission from Uekert et al.,^38^ copyright@2019, American Chemical Society. Reproduced or adapted with permission from Du et al.,^39^ copyright@2022, American Chemical Society. Reproduced or adapted with permission from Kang et al.,^40^ copyright@2023, American Chemical Society. Reproduced or adapted with permission from Zhang et al.^41^ Reproduced under the terms of the CC-BY license. Reproduced or adapted with permission from Yue et al.,^42^ copyright@2024, Wiley. Reproduced or adapted with permission from Han et al.,^43^ copyright@2025, Wiley.

### Traditional photocatalytic CO_2_ reduction

The photocatalyst is the core component of a photocatalytic system. For conventional CO_2_ reduction, the conduction band (CB) minimum must be more negative than the reduction potential of CO_2_, while the valence band (VB) maximum must be more positive than the water oxidation potential (1.23 V vs. NHE (normal hydrogen electrode) at pH = 0).^45^^,^^46^^,^^47^ As a result, only a limited number of semiconductors satisfy both thermodynamic requirements (Figure 4A). In practice, most photocatalysts fail to simultaneously meet these criteria, and even for those that do, the sluggish kinetics of the four-electron water oxidation half-reaction severely limit the overall efficiency. The most common strategy to circumvent this bottleneck is the addition of organic sacrificial agents (e.g., triethanolamine) to rapidly consume photogenerated holes, thereby suppressing charge recombination and enhancing the electron availability for CO_2_ reduction. However, this approach increases operational costs and introduces secondary pollution. In contrast, plastic photoreforming offers a distinct advantage: the oxidation potential required for plastic degradation is substantially lower than that for water oxidation. This allows the use of semiconductors with shallower VB positions, significantly broadening the pool of accessible photocatalysts and eliminating the need for external sacrificial agents.^48^

**Figure 4:**
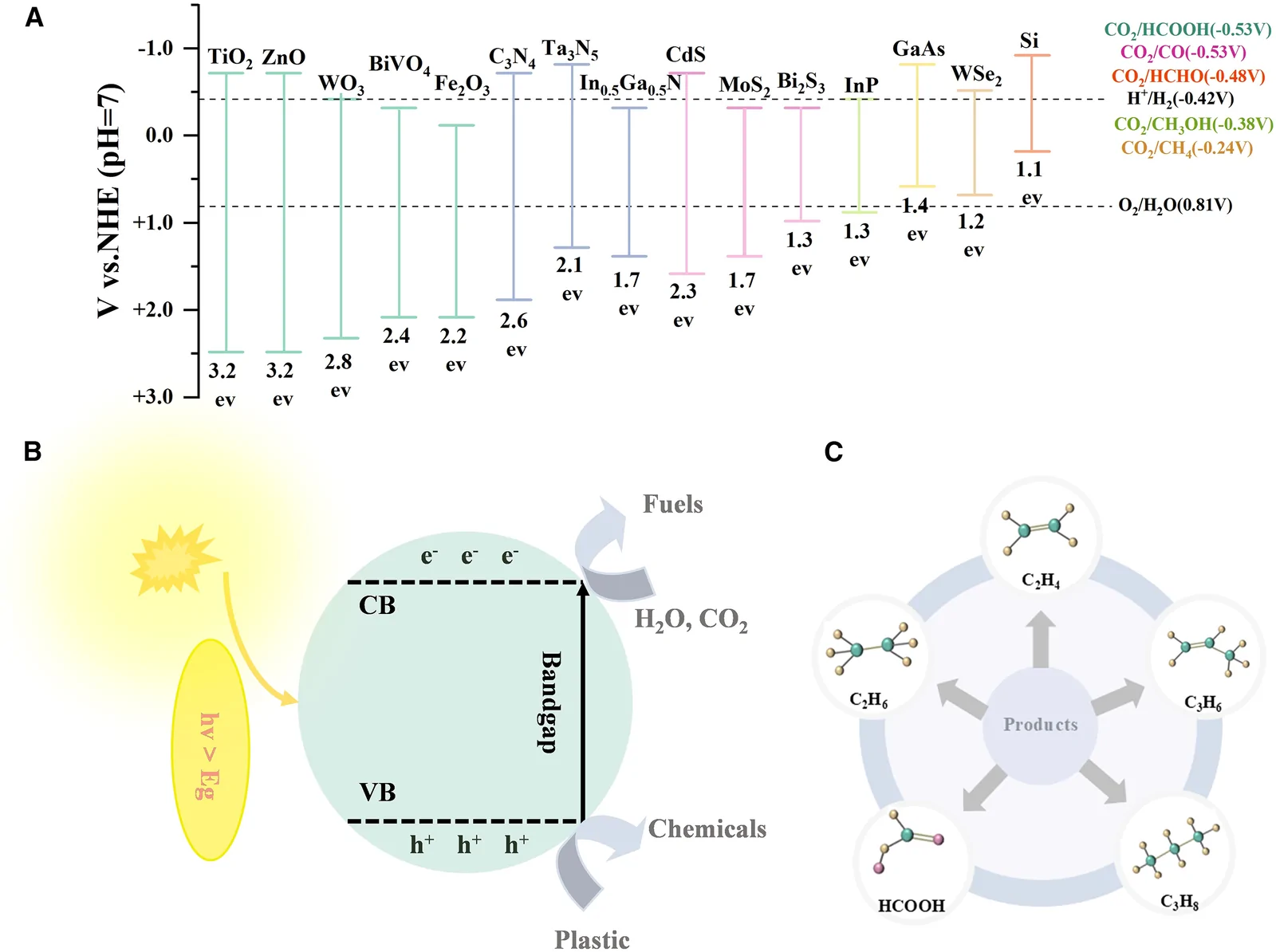
Photocatalyst band structures and reaction mechanism (A) Potential scaling of band gaps and band edge positions of various semiconductors. (B) Mechanism of plastic photoreforming. (C) Classification of photoreforming products.

### Plastic photoreforming for CO_2_ reduction

In conventional photocatalytic CO_2_ reduction systems, CO_2_ is supplied exclusively from external sources, while water serves both as a proton donor and as a hole-consuming medium. The overall efficiency is severely constrained by the water oxidation half-reaction, which involves a four-electron transfer process and must overcome a thermodynamic barrier of approximately 1.23 V along with a significant kinetic overpotential. The target products are primarily C_1_ fuels such as CO, CH_4_, and HCOOH.^49^^,^^50^^,^^51^^,^^52^ In contrast, in plastic photoreforming systems, the carbon source can be derived from the decomposition of plastics. Polymer chains are broken down into small-molecule organic acids under the action of photo-generated holes or reactive oxygen species (e.g., ·OH, ·O_2_^−^), accompanied by a substantially accelerated hole consumption rate (Figure 4B). This rapid hole scavenging effectively suppresses charge recombination and prolongs the lifetime of photogenerated electrons available for CO_2_ reduction. Consequently, the range of target products is broader: the reduction side yields fuels such as CO and CH_4_, while the oxidation side simultaneously produces high-value organic chemicals (Figure 4C). This dual functionality positions plastic photoreforming as a promising platform for simultaneous waste valorization and CO_2_ utilization, which will be discussed in detail with representative examples in the following section. In addition to serving as a secondary carbon source, plastics can also act as electron donors in coupled systems. In traditional photocatalytic CO_2_ reduction, electrons are generated only by light-excited semiconductors. However, in photoreforming, the introduction of plastics creates an additional electron supply pathway. Specifically, when the C–H/C–C bonds in plastic molecular chains break at the catalyst’s active sites, electrons are released and injected into the catalyst, further participating in CO_2_ activation. This not only reduces the overpotential of CO_2_ reduction but also suppresses the recombination of photogenerated carriers. Therefore, plastics possess the dual identities of both “electron donor” and “carbon source.”

## Upgrading photocatalysis: Plastic coupling catalytic reactions

### Photocatalytic CO_2_ reduction reaction

Photocatalytic CO_2_ reduction primarily occurs in aqueous solutions and involves the following six steps^53^^,^^54^^,^^55^^,^^56^^,^^57^^,^^58^: (1) photoexcitation, in which the photocatalyst absorbs photon energy upon light irradiation to initiate the photoactivation process; (2) generation of electron-hole pairs, with electrons and holes generated in the CB and VB of the semiconductor, respectively; (3) migration of electron-hole pairs, during which photogenerated electrons and holes migrate to the surface of the photocatalyst; (4) CO_2_ adsorption and activation, where CO_2_ molecules adsorb onto the catalyst surface and accept electrons for subsequent reduction; (5) redox reactions, in which the adsorbed CO_2_ is reduced to products such as CO, CH_3_OH, and CH_4_, while H_2_O is oxidized to O_2_ and H^+^; and (6) desorption of products, where the products are rapidly desorbed from the catalyst surface to enable subsequent redox cycles. Among these steps, CO_2_ interfacial adsorption, activation, and electron transfer are the most critical steps in the overall reaction, determining both product selectivity and catalytic activity.

Xu et al. constructed a composite catalytic system comprising Re-bpy and a porous heterogeneous copper porphyrin-triazine framework (PTF(Cu)) for the photocatalytic reduction of CO_2_ to ethylene.^59^ This system achieved an ethylene production rate of 73.2 μmol g^−1^ h^−1^ with a selectivity of 25% (Figures 5A and 5B). Mechanistic studies revealed that the Re–∗CO species generated on Re-bpy undergo cross-metallic C–C coupling with the Cu-∗CO species adsorbed at the single-atom Cu sites of PTF(Cu), forming the key Re–CO–CO–Cu intermediate that enables ethylene production. The efficient and substantial *in situ* supply of ∗CO intermediates by Re–bpy, the shortened inter-site distance facilitated by the porous confinement effect, and the enhanced photogenerated charge separation are identified as the primary factors contributing to the improved photocatalytic activity (Figure 5C).

**Figure 5:**
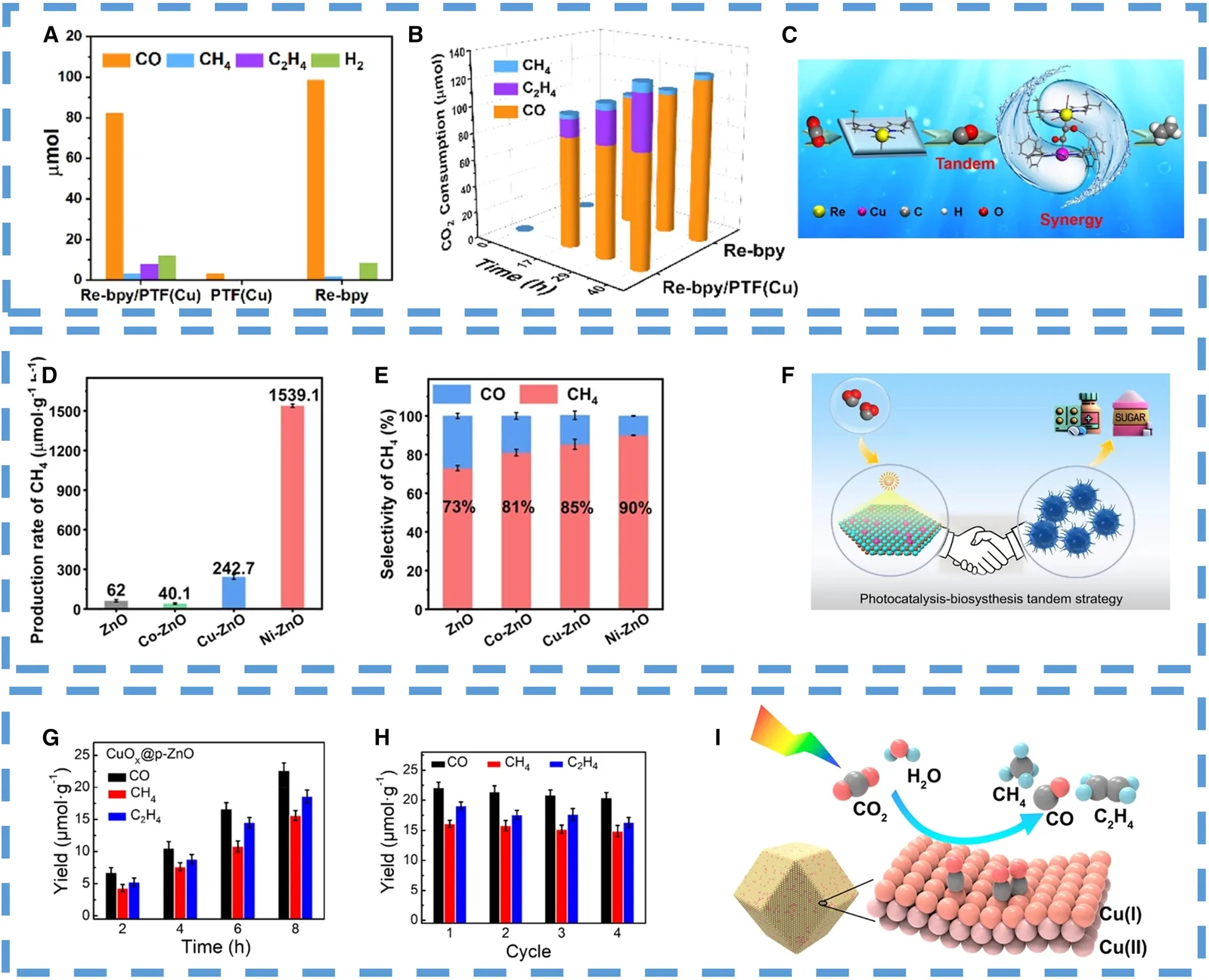
Photocatalytic CO_2_ reduction reaction (A) Comparison of CO_2_ reduction product yields in different photocatalytic systems. (B) 3D diagram of CO_2_ consumption over time in a series system. (C) Schematic diagram of Re-Cu series synergistic catalysis. Reproduced or adapted with permission from Xu et al.,^59^ copyright@2023, American Chemical Society. (D) CH_4_ yield of metal-doping ZnO. (E) Selectivity of products from ZnO with different metal-doping. (F) Reaction pathways for CO_2_ reduction. Reproduced or adapted with permission from Yu et al.,^60^ copyright@2025, Wiley. (G) Yield of CuO_x_@p-ZnO. (H) Catalyst cycling stability. (I) Reaction mechanism. Reproduced or adapted with permission from Wang et al.,^61^ copyright@2021, American Chemical Society.

In a different approach that integrates photocatalysis with biosynthesis, Yu and colleagues employed Ni-doped two-dimensional ZnO nanosheets (Ni-ZnO) to convert CO_2_ into value-added chemicals.^60^ This system delivered a CH_4_ production rate of 1539.1 μmol g^−1^ h^−1^ with 90% selectivity (Figures 5D and 5E). The core of this process lies in the Ni-ZnO configuration, characterized by a Zn^δ+^-*O*-Ni^β+^ structural motif, which effectively lowers the energy barrier of the rate-determining step and thereby accelerates the multi-electron transfer required for CO_2_–to–CH_4_ conversion (Figure 5F).

Alternatively, Wang et al. synthesized a composite photocatalyst, CuO_x_@p-ZnO, via high-temperature calcination of Cu-doped ZIF-8 as the precursor, wherein CuO_x_ species are uniformly dispersed within polycrystalline ZnO.^61^ In the presence of triethylamine as a sacrificial agent, the C_2_H_4_ yield was enhanced to 22.3 μmol g^−1^ h^−1^ (Figures 5G and 5H). The composite catalyst exploits a photoinduced self-activation process to form a mixed-valence interface consisting of a Cu^+^-enriched surface layer and a CuO bulk phase, which effectively overcomes the bottlenecks of facile CO desorption and high C–C coupling energy barriers commonly encountered in photocatalytic systems. Moreover, the OC–COH intermediate was successfully detected using *in situ* characterization techniques, providing direct evidence for the reaction pathway (Figure 5I).

In traditional photocatalytic CO_2_ reduction reactions, the performance ceiling and product branching pathways are governed by the adsorption behavior at the catalyst–CO_2_ interface, the ease of molecular activation, and the efficiency of interfacial electron transfer. The sluggish water oxidation process consumes a significant portion of photogenerated charges, reducing the number of effective electrons available for CO_2_ reduction. In contrast, a photoreforming system that uses waste plastic as the hole-capturing medium can rapidly consume photogenerated holes, thereby optimizing interfacial charge transport and enhancing electron utilization. Based on this advantage, the following section will provide a detailed analysis of the plastic photoreforming systems coupled with CO_2_ reduction.

### Plastic-coupling photocatalytic CO_2_ reduction

Unlike traditional semiconductor-based photocatalytic CO_2_ reduction systems, the hole-consumption pathway undergoes a fundamental change in plastic photoreforming CO_2_ reduction systems. Traditional systems rely on the oxidation of water molecules to consume photogenerated holes, but the high kinetic barrier of water oxidation results in slow reaction rates. In contrast, plastic photoreforming systems use waste plastic macromolecules as a hole-scavenging substrate in place of water. Photogenerated holes migrating to the catalyst surface can rapidly oxidize the plastic backbone, simultaneously achieving plastic degradation and resource recovery while suppressing electron-hole pair recombination and significantly improving electron utilization.^62^^,^^63^^,^^64^^,^^65^^,^^66^

For example, Xu et al. employed Co-doped Ga_2_O_3_ nanosheets (Co-Ga_2_O_3_) as a photocatalytic material to achieve simultaneous degradation of plastic waste and green production of CO and H_2_ in an aqueous phase under ambient temperature and pressure (Figure 6A).^67^ The Co–Ga_2_O_3_ catalyst efficiently degraded three types of commercial plastics, namely, PE plastic bags, PP lunch boxes, and PET bottles. Taking PE as a representative substrate, the H_2_ and CO evolution rates reached 647.8 μmol g^−1^ h^−1^ and 158.3 μmol g^−1^ h^−1^, respectively, which are 1.6 and 1.9 times higher than those of pristine Ga_2_O_3_ (Figure 6B). Concurrent detection of CO_2_ as an intermediate confirmed that the generated CO originated from the secondary photoreduction of CO_2_ derived from plastic degradation. A series of characterizations unequivocally demonstrated that H_2_ was exclusively produced from water reduction, while both CO_2_ and CO were generated through the synergistic oxidation of plastics by hydroxyl radicals and molecular oxygen. In the absence of plastics, only H_2_ evolution was observed with no CO production, further corroborating the proposed reaction pathway. Zhang et al. developed a series of Zn_x_Cd_1-x_S catalysts with tunable band structures for the concurrent conversion of CO_2_ and PET into syngas and acetate.^68^ Under 4 h of illumination, the PET conversion reached 75.5%. In the optimal Zn_0.8_Cd_0.2_S system, the acetate production rate was 202.5 μmol g^−1^ h^−1^, and the total syngas evolution rate was 1.7 mmol g^−1^ h^−1^. Notably, the H_2_/CO ratio of syngas could be finely adjusted over a wide range, and the ideal 3:2 ratio desirable for alkane synthesis could be stably achieved (Figures 6C and 6D). Mechanistic investigations revealed that the rapid consumption of valence-band holes by PET effectively suppressed electron-hole recombination, thereby increasing the number of available electrons for CO_2_ activation. Furthermore, tuning the Zn/Cd ratio enabled precise control over CO_2_ adsorption strength, intermediate stability, and CO desorption capability, ultimately achieving accurate regulation of the syngas composition (Figure 6E). In another study, Zhang et al. introduced Pd species onto TiO_2_ to enable the highly selective conversion of PE plastic waste into C_2_H_4_ and propionic acid under ambient conditions (Figure 6F).^41^ The Pd_1_-TiO_2_ catalyst exhibited a C_2_ hydrocarbon production rate of 1033 μmol g_cat_^−1^ h^−1^, which is 50 times and 5.4 times higher than those of bare TiO_2_ and Pd_n_-TiO_2_, respectively. Moreover, the system demonstrated an exceptional selectivity of 98.8% toward propionic acid, with a yield of 493.3 μmol after 3 h of reaction (Figures 6G and 6H). Comprehensive characterization confirmed the dual role of atomically dispersed Pd species: they not only serve as electron acceptors that promote charge separation and transfer for efficient photoredox processes, but also act as mediators that stabilize the intermediates involved in the selective decarboxylation pathway.

**Figure 6:**
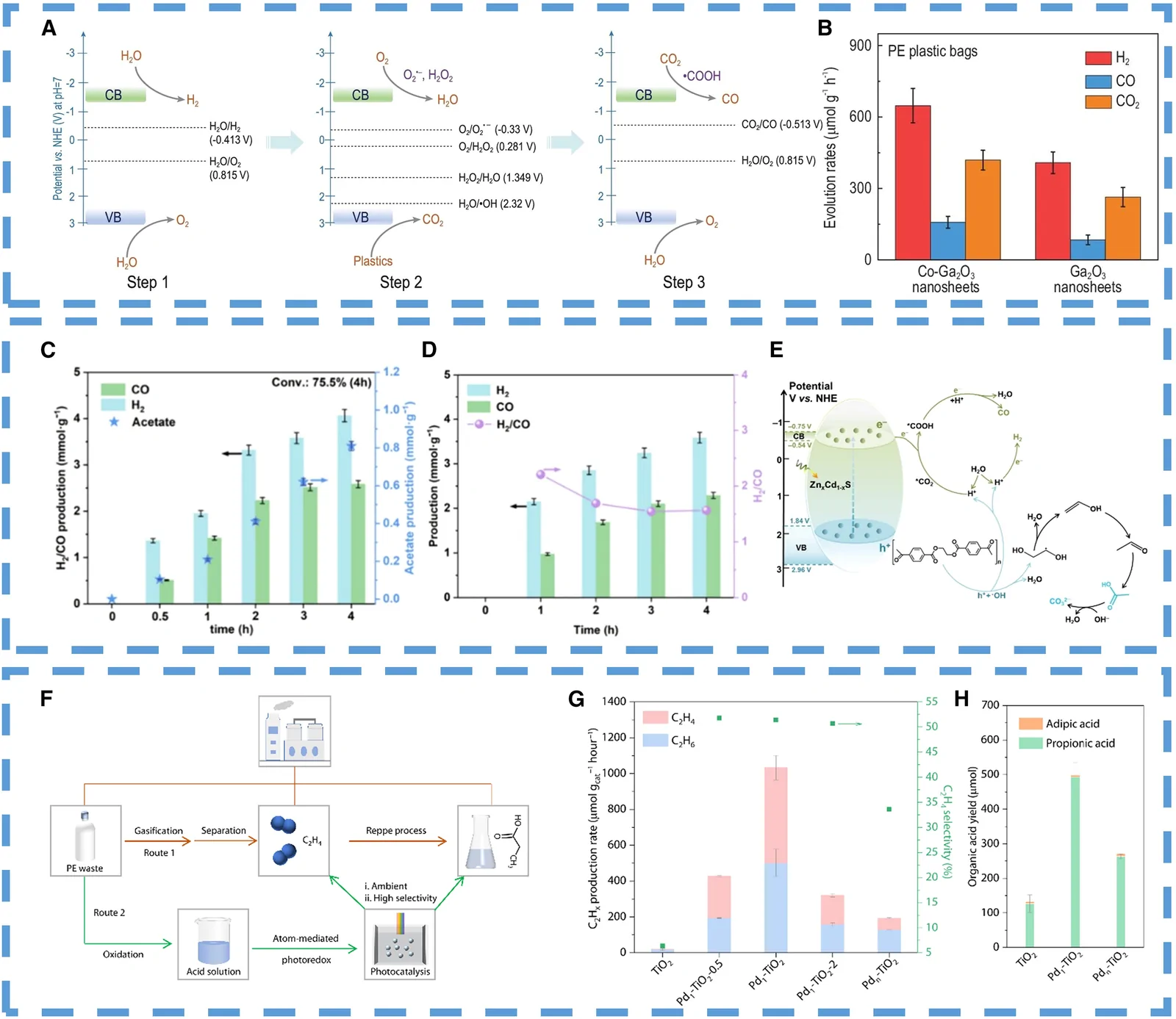
Plastic-coupling photocatalytic CO_2_ reduction (A) Three-step band mechanism of plastic photoreforming. (B) Gas evolution rates over Ga-based nanosheets. Reproduced or adapted with permission from Xu et al.,^67^ copyright@2022, Oxford University Press. (C and D) Time-resolved H_2_/CO production and H_2_/CO ratio on Zn_0.2_CdS_0.8_. (E) Reaction mechanism. Reproduced or adapted with permission from Zhang et al.,^68^ copyright@2025, American Chemical Society. (F) Two valorization routes for waste PE. (G) C_2_ hydrocarbon activity over Pd/TiO_2_. (H) Organic acid yields on Pd-modified TiO_2_. Reproduced or adapted with permission from Zhang et al.^41^ Reproduced under the terms of the CC-BY license.

In addition, a number of analogous studies have been reported. Jiao et al. designed a single-layer, single-unit-cell Nb_2_O_5_ photocatalyst that operates under simulated natural conditions: ambient temperature and pressure, pure water, air atmosphere, and without any sacrificial agents.^69^ This system establishes a two-step sequential C-C bond cleavage/coupling pathway, enabling the complete degradation of various waste plastics, including PE, PP, and PVC (polyvinyl chloride), with concurrent selective conversion to acetic acid. The catalyst achieved 100% photodegradation of PE within 40 h. Beyond the dedicated photodegradation of PE to CO_2_, the generated CO_2_ was further selectively photoreduced to CH_3_COOH, demonstrating an integrated degradation-upcycling process (Figures 7A–7C). Nguyen et al. employed BaTiNbTaZnO_9_ as a photocatalyst to simultaneously achieve CO_2_ reduction and PET plastic conversion under light irradiation.^70^ The evolution rates of CO and CH_4_ reached 116.1 and 5.9 μmol h^−1^ g^−1^, respectively, with a CO selectivity of 95%. The exceptionally high selectivity was primarily attributed to the presence of Zn, which exhibits weak CO binding affinity, thereby favoring CO release over its further reduction (Figures 7D–7F). Li et al.^14^ reported a tandem process utilizing a dinuclear Ru-based molecular heterogeneous catalyst for the concurrent degradation of waste PET plastics and photoreduction of CO_2_. In this system, the Ru center is responsible for photooxidation of the plastic monomers, while the Re site serves exclusively for CO_2_ reduction. The spatial separation of the two active sites enables effective redox compartmentalization, resulting in significantly enhanced product selectivity and turnover numbers (Figures 7G–7I).

**Figure 7:**
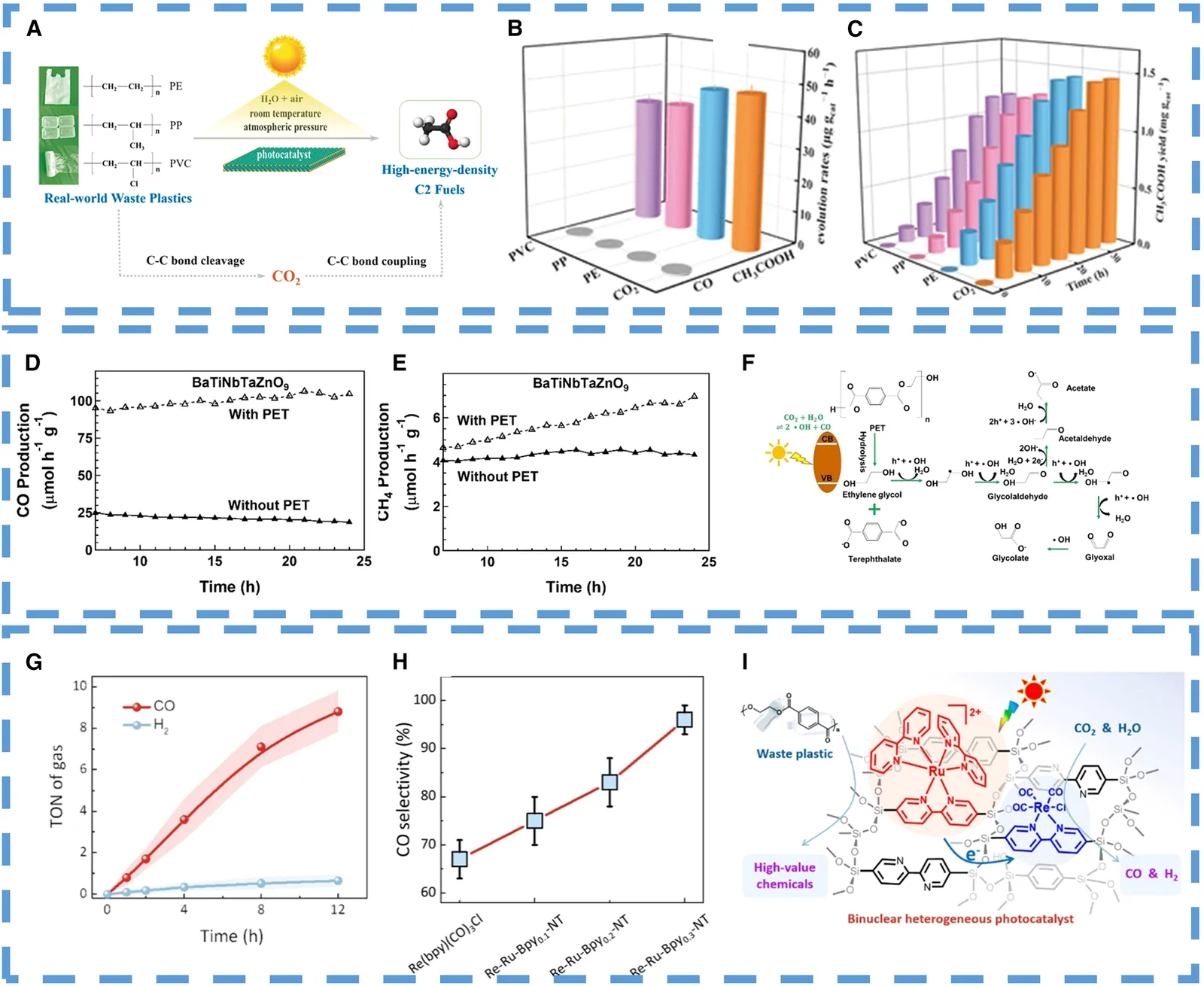
Plastic-coupling photocatalytic CO_2_ reduction (A) Overall route for plastic-to-C_2_ fuel conversion. (B) Product evolution rates of different plastics. (C) Time-dependent product profiles. Reproduced or adapted with permission from Jiao et al.,^69^ copyright@2020, Wiley. (D and E) Long-term CO/CH_4_ stability of BaTiNbTaZnO_9_ with/without PET. (F) Reaction mechanism. Reproduced or adapted with permission from Nguyen et al.,^70^ copyright@2026 Wiley. (G) TON evolution of gaseous products. (H) CO selectivity of catalysts. (I) Dual-functional mechanism of photocatalyst. Reproduced or adapted with permission from Li and Zhang,^14^ copyright@2024, American Chemical Society.

The studies above demonstrate the feasibility of a photo-driven system that couples plastic reforming with CO_2_ reduction, confirming that plastic oxidation and CO_2_ reduction can be achieved solely by using light energy to excite a semiconductor catalyst. However, this pure suspended-phase photocatalytic system without an external bias still faces universal development bottlenecks: severe bulk and surface charge carrier recombination and inherent reaction rate limitations, which restrict further improvements in CO_2_ conversion yield, carbon product selectivity, and industrial scalability. To tackle these bottlenecks, introducing an external bias to construct a photoelectrocatalytic system is an effective approach to address the above limitations.

### Plastic-coupling photoelectrocatalytic CO_2_ reduction

Benefiting from the controllable external electric field, the integrated photoelectrocatalytic system can precisely regulate the applied bias voltage, electrolyte pH, and electrode potential, thereby finely tuning the formation of key intermediates such as ^∗^COOH. For instance, in the dual-electrode separated electrolytic cell, the anode drives the selective oxidation of ethylene glycol (EG), the hydrolysis product of PET, while the cathode is used for CO_2_ reduction. The spatially separated redox reactions avoid mutual interference.^71^^,^^72^^,^^73^^,^^74^^,^^75^ Such a controllable reaction system effectively improves catalytic efficiency and product selectivity, exhibiting great potential for scalable practical applications.

Li et al. constructed a ternary photoanode comprising ultrathin carbon-layer-modified Ni(OH)_2_/C/TiO_2_, which was paired with a Si-based CuBi_300_ photocathode to assemble an unbiased photoelectrochemical tandem cell.^72^ This integrated system enabled the simultaneous and directional conversion of PET plastic oxidation and CO_2_ reduction to formate, achieving FE of 171.8% at 0 V and FE_max_ of 192.2% at 0.3 V. The synergistic effect of the applied electric field and interfacial modulation by the carbon layer effectively suppressed carrier recombination at the source and eliminated excessive oxidation side reactions caused by hydroxyl radicals, thereby substantially improving product selectivity and conversion efficiency (Figures 8A–8C). Wang et al. designed a bifunctional copper hydroxide-based photoelectrocatalytic system that couples the ethylene glycol oxidation reaction with CO_2_ reduction,^73^ enabling the simultaneous upcycling of waste plastics and CO_2_ conversion into two types of high-value carbon chemicals, namely formate and ethylene, respectively (Figures 8D–8G). Liu et al. devised a self-standing organic-inorganic photoelectrocatalytic reaction (PEC) tandem device capable of co-utilizing CO_2_ and PET for sustainable formate production, demonstrating excellent performance. During the reaction process, PET oxidation consumes photogenerated holes as a substitute for the energy-intensive oxygen evolution reaction (OER), while CO_2_ reduction consumes electrons, leading to a significant reduction in overall energy loss.^74^ Moreover, the unification of both anodic and cathodic products into a single product, formate, eliminates the need for multi-component separation procedures, greatly simplifying downstream processing (Figures 8H–8J). The key parameters and performance metrics of these representative studies are summarized in Table 1.

**Figure 8:**
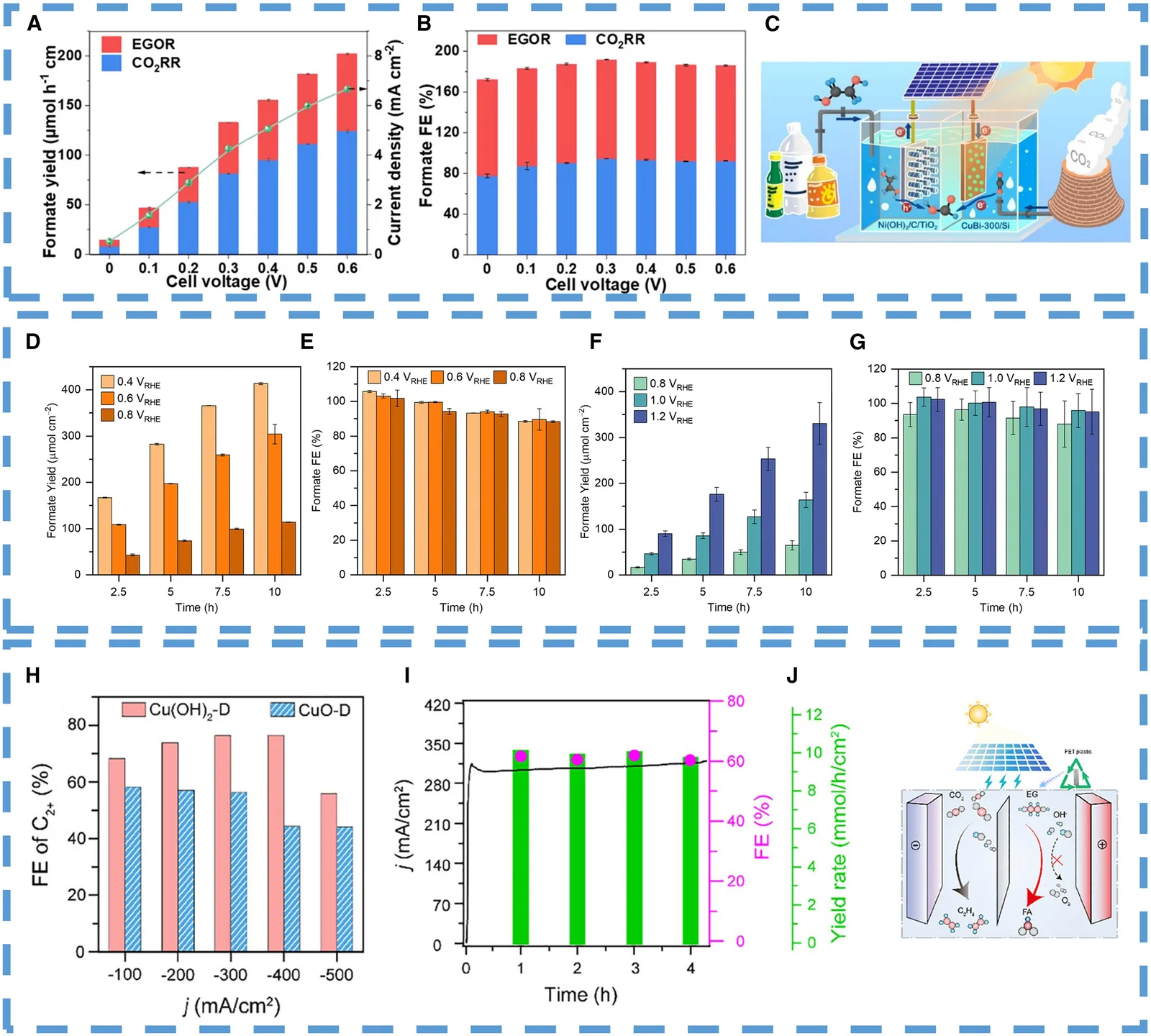
Plastic-coupling photoelectrocatalytic CO_2_ reduction (A) Formate yield. (B) FE. (C) Schematic of photodriven co-electrolysis reactor. Reproduced or adapted with permission from Li et al.,^72^ copyright@2025, Wiley. (D and E) Time-resolved formate yield and FE at low potentials within 10 h. (F and G) Time-dependent formate yield and FE at high potentials over 10 h. Reproduced or adapted with permission from Wang et al.,^73^ copyright@2025, Wiley. (H) C_2+_ FE of two Cu-derived catalysts. (I) Long-term stability. (J) Microscopic pathway of photoelectric co-electrolysis. Reproduced or adapted with permission from Liu et al.,^74^ copyright@2025, Royal Society of Chemistry.

**Table 1:** Research summary: Plastic reforming for CO_2_ reduction

Catalyst	Reaction type	Reaction conditions	Products	Product activity	Plastics	Reference
Co–Ga_2_O_3_	PC	AM 1.5 G 100 mW/cm^2^	CO	158.3 μmol g^−1^·h^−1^	PE	Xu et al.^67^
Zn_0.8_Cd_0.2_S	PC	300 W xenon lamp	acetate	202.5 μmol g^−1^·h^−1^	PET	Zhang et al.^68^
Pd1-TiO_2_	PC	365 nm high-power LED	C_2_ hydrocarbon	1033 μmol g^−1^ h^−1^	PE	Zhang et al.^41^
Nb_2_O_5_	PC	300 W xenon lamp	CH_3_COOH	47.4 mg g_cat_^−1^·h^−1^	PE	Jiao et al.^69^
BaTiNbTa-ZnO_9_	PC	400 W mercury lamp	CO CH_4_	116.1 μmol g^−1^ h^−1^ 5.9 μmol g^−1^ h^−1^	PET	Nguyen et al.^70^
Re-Ru-BPy-NT	PC	300 W xenon lamp	HCOOH CO	–	PET	Li and Zhang^14^
ZnS/Ga_2_O_3_	PC	AM 1.5 G 100 mW/cm^2^	CO	27.32 μmol g^−1^ h^−1^	PE	Xu et al.^71^
Ni(OH)_2_/C/TiO_2_	PEC	light 0.3 V	formate HCOOH	14.3 μmol cm^−2^ h^−1^	PET	Li et al.^72^
OPV	PEC	AM1.5G 0 V vs. RHE 0.37 mA	formate	11 mmol cm^−2^ h^−1^	PET	Wang et al.^73^
Cu(OH)_2_/Cu(OH)_2_-D	PEC	AM 1.5 G 302.7 mA/cm^−2^	formate C_2_H_4_	4.72 mmol cm^−2^·h^−1^ 9.65 mmol cm^−2^·h^−1^	PET	Liu et al.^74^
Ti-Fe_2_O_3_/Ni(OH)_X_	PEC	AM 1.5 G	formic acid	0.277 mol m^−2^ h^−1^	PET	Li et al.^75^

PC, photocatalytic reaction; PEC, photoelectrocatalytic reaction.

## Conclusion and outlook

This review provides a comprehensive analysis of the reaction processes, underlying mechanisms, and existing research challenges associated with plastic photoreforming coupled with CO_2_ reduction in comparison to conventional CO_2_ photoreduction. The historical evolution of photoreforming combined with CO_2_ reduction is systematically surveyed, tracing the developmental trajectory of this emerging field. Beyond syngas production, this review also highlights the generation of high-value chemical products derived from plastic photoreforming processes. Particular emphasis is placed on recent advances in photoelectrocatalytic coupled systems, where three representative routes, including conventional photocatalytic CO_2_ reduction, plastic-coupled photoreforming, and integrated photoelectroreforming are compared. This plastic-coupled strategy enables waste plastics to act as both carbon feedstock and reaction substrates to produce CO and value-added chemicals, holding great promise for carbon neutrality. From the perspective of practical feasibility, the pure photocatalytic system features simple device configuration without external bias, yet it suffers from severe charge-carrier recombination and low throughput. Plastic-coupled photocatalytic photoreforming realizes dual utilization of waste plastics as electron donors and carbon sources, but it is limited by insufficient catalyst stability and moderate reaction rates. The integrated photoelectrocatalytic system enables flexible potential regulation and spatial separation of redox reactions for improved performance, at the expense of more complex assembly and higher system costs. Despite considerable progress, multiple bottlenecks still hinder the practical deployment of plastic photoreforming coupled with CO_2_ reduction. Based on the above research advances, we outline future research directions to inspire further breakthroughs in this interdisciplinary field (Figure 9):

**Figure 9:**
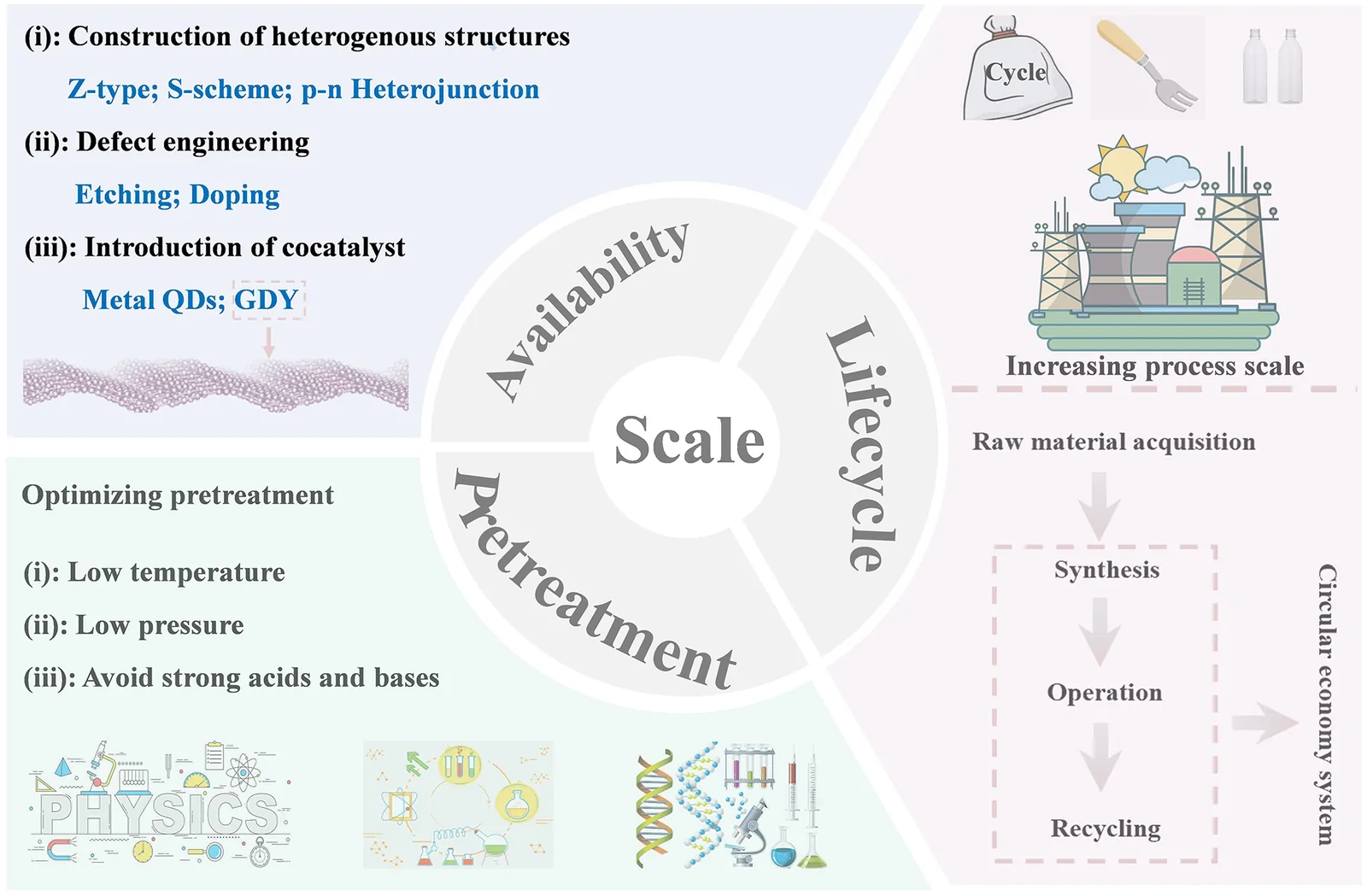
Challenges and recommended future research directions for photocatalytic upgrading of plastic waste

This review provides a comprehensive analysis of the reaction processes, underlying mechanisms, and existing research challenges associated with plastic photoreforming coupled with CO_2_ reduction in comparison to conventional CO_2_ photoreduction. The historical evolution of photoreforming combined with CO_2_ reduction is systematically surveyed, tracing the developmental trajectory of this emerging field. Beyond syngas production, this review also highlights the generation of high-value chemical products derived from plastic photoreforming processes. Particular emphasis is placed on recent advances in photoelectrocatalytic coupled systems, encompassing comparative performance evaluations of conventional photocatalytic CO_2_ reduction, plastic photoreforming coupled with CO_2_ reduction, and integrated plastic photoelectroreforming with CO_2_ reduction. The prospects of utilizing plastic-coupled reactions in photocatalytic systems are underscored, given that plastics can serve as a source of CO_2_ while concurrently yielding CO and other high-value chemicals, thereby positioning this technology as a pivotal contributor toward achieving carbon-neutrality goals. Despite the substantial progress made in plastic photoreforming coupled with CO_2_ reduction, it must be acknowledged that numerous issues and challenges remain to be addressed. On the basis of the foregoing research advancements, we outline potential directions for future research and development, with the aim of guiding further breakthroughs in this interdisciplinary field (Figure 9).

(1) Photocatalyst upgrading: the catalytic core of the plastic photoreforming coupled with CO_2_ reduction system lies in the photoelectric conversion process of the semiconductor photocatalyst.^76^^,^^77^^,^^78^ As the central component, the photocatalyst’s band structure, interfacial active sites, and charge-carrier migration capability directly determine the conversion efficiency and product selectivity of the coupled reaction. In this context, the rational design of highly efficient and stable catalysts represents an urgent challenge. To achieve efficient utilization of photocatalysts, researchers have proposed various strategies, including but not limited to heterojunction construction (Z-scheme, S-scheme, and p-n heterojunctions),^79^ defect engineering (such as etching and doping),^79^ and the introduction of cocatalysts (metals, graphdiyne (GDY), and others).^80^^,^^81^^,^^82^ Through these approaches, the photocatalytic activity can be significantly enhanced, thereby promoting efficient reaction progress. Shi et al.^83^ constructed a CoS_1.097_/ZnIn_2_S_4_ heterojunction. The optimized catalyst achieved a CH_4_ generation rate of 24.16 μmol g^−1^ h^−1^ under visible light irradiation and without sacrificial agents. The heterojunction interface induced interface charge redistribution, the formation of built-in electric field, and enhanced the occupation degree of Co 3d orbitals and the shift of the d-band center, thereby achieving electronic structure regulation. In addition, You et al.^84^ summarized the promoting effect of co-catalysts on photocatalytic CO_2_ reduction, and Xiao et al.^85^ introduced the application progress of some defect-mediated catalysts in CO_2_ reduction. All these indicate that the introduction of co-catalysis and defect engineering can not only increase the active sites of the reaction, effectively reduce the energy barrier required for the reaction, but also more fully absorb light energy and more effectively utilize charges. Furthermore, continued efforts should be devoted to the development of novel photocatalysts that maximize the contact area between the plastic substrates and the catalyst surface, which is essential for facilitating reaction kinetics and improving overall performance.
(2) Pretreatment optimization: prior to photoreforming, appropriate pretreatment of plastic feedstocks is typically required. Conventional pretreatment methods generally fall into physical and chemical categories; however, both approaches suffer from certain drawbacks, including high-temperature and high-pressure conditions, the use of strongly acidic or alkaline reaction media, and the generation of hazardous byproducts. In contrast, biological pretreatment represents a more environmentally benign and sustainable alternative, wherein enzymes or microorganisms are employed to modify the plastic substrates, thereby circumventing the environmental hazards associated with traditional methods. Notably, enzymes and microorganisms have already been widely applied in various photocatalytic systems. Wang et al.^86^ utilized the extract of Pichia pastoris GS115 to prepare a copper-doped LaCoO_3_ perovskite photocatalyst through a microbial-assisted method. This material exhibited a hydrogen production rate of 1.13 mmol h^−1^ g^−1^ during the photocatalytic degradation of formaldehyde. Li et al.^87^ coupled ZnO/ZnS nanoparticles with the capsule-shaped red bacterium SB 1003. They found that the photogenerated electrons produced by ZnO/ZnS could be effectively captured by this strain, which not only activated the nitrogenase activity but also significantly improved the photosynthetic hydrogen production efficiency. Looking forward, the integration of microbial pretreatment with photoreforming processes holds considerable promise, as it not only enables mild and eco-friendly plastic pretreatment but also facilitates the concurrent production of high-value chemicals through synergistic biocatalytic and photocatalytic pathways.
(3) Process scale-up: the technology of plastic photoreforming coupled with CO_2_ reduction enables the simultaneous valorization of waste plastics and the recycling of CO_2_. Compared with single-component catalytic systems, it offers distinct advantages, including lower energy consumption, reduced raw material costs, and enhanced dual-carbon mitigation benefits, thereby holding substantial promise for industrial implementation. Nevertheless, scale-up remains constrained by several bottlenecks, such as the high cost of photocatalysts, limitations in reactor design, catalyst poisoning induced by mixed-plastic feedstocks, and difficulties in product separation. To address these challenges, future efforts should focus on the development of an integrated technological chain encompassing catalyst preparation, plastic feedstock supply, photocatalytic reaction, and end-of-life treatment of products and residues.^88^^,^^89^ In parallel, life cycle assessment (LCA) should be employed to systematically optimize the overall energy efficiency and environmental burden of the entire system. However, at present, plastic photoreforming coupled with CO_2_ reduction remains predominantly at the laboratory scale, and the availability of reliable database inventories for scaled-up continuous reactions and industrial-level CO_2_ capture is still insufficient, leading to considerable uncertainty in LCA evaluation. The design and pre-treatment of the catalyst have been discussed in detail above, demonstrating their feasibility. Photocatalytic reactions can be scaled up from the laboratory to industrialization through experiments, such as plate-type reactors, which have already been preliminarily applied in the field of photocatalysis; moreover, with the rapid development of artificial intelligence (AI), the end treatment can use AI to identify and classify residues. Through AI, the various processes mentioned earlier can be connected in series, analyzing various data, identifying the key influencing parameters in the production process, and optimizing the catalytic process, thereby overcoming many challenges in the large-scale process and having great application potential.

In summary, plastic photoreforming coupled with CO_2_ reduction has far-reaching significance, yet it presents both tremendous opportunities and challenges. Future research progress will depend on catalyst upgrades, in-depth mechanistic studies, and the careful design of next-generation catalytic systems.

## Acknowledgments

This work was financially supported by the 10.13039/501100001809National Science Foundation of China (22502241), Guangdong Provincial Excellent Young Scholars Program (2026A1515030052), and Shenzhen Science and Technology Program (JCYJ20240813151113018).

## Author contributions

Y.Z. and Y.G., conceptualization, investigation, writing – original draft, and formal analysis. G.H., supervision, writing – review and editing, and funding acquisition. All authors read and approved the manuscript.

## Declaration of interests

The authors declare no competing interests.
